# Evolution spectaculaire d’un pemphigus végétant réfractaire aux thérapies conventionnelles sous Rituximab: à propos d’un cas

**DOI:** 10.11604/pamj.2019.32.101.16743

**Published:** 2019-03-04

**Authors:** Younes Barbach, Hanane Baybay, Samia Mrabat, Mohammed Chaouche, Sara Elloudi, Fatima Zahra Mernissi

**Affiliations:** 1 Service de Dermatologie, CHU Hassan II, Fès, Maroc

**Keywords:** Pemphigus végétant, rare, rituximab, Pemphigus vegetans, rare, rituximab

## Abstract

Le pemphigus végétant est une variété rare de pemphigus. Il n'y représente que 2%. Plusieurs traitements sont de mise pour traiter cette entité, on retrouve les corticostéroïdes topiques et/ou oraux en 1^ère^ intension, les immunosuppresseurs tels que l'Azathioprine, la Cyclosporine, le Méthotrexate, le Cyclophosphamide et le Mycophénolate mofétil pour remédier au effet secondaires des stéroides. Certains cas restent réfractaires à tous ces traitements, l'utilisation du Rituximab a fait révolutionner la prise en charge du pemphigus, en particulier sa forme végétante. Nous rapportons le cas d'une patiente de 42ans, admise pour prise en charge d'un pemphigus végétant confirmé histologiquement et mise initialement sous corticothérapie en association avec immunosuppresseur sans amélioration, puis fut mise sous Rituximab avec une évolution spectaculaire.

## Abstract (en)

Pemphigus vegetans is a rare form of pemphigus. It accounts for only 2%. There is a broad range of effective options available to treat this disease: topical and/or oral corticosteroids as first-line treatment, immunosuppressants such as Azathioprine, Cyclosporine, Methotrexate, Cyclophosphamide and Mycophenolate mofetil to reduce the side effects of steroids. Some patients are resistant to all these treatments. Rituximab has revolutionized the treatment of pemphigus, pemphigus vegetans in particular. We here report the case of a 42-year old female patient, admitted with pemphigus vegetans confirmed by histology. She initially received corticosteroid therapy associated with immunosuppressive therapy without improvement. Then she was treated with Rituximab and had an unexpected positive outcome.

## Introduction

Le pemphigus végétant est considéré comme une forme rare du pemphigus vulgaire, caractérisée par une atteinte des plis et un aspect végétant des lésions par endroit. Nous rapportons un cas avec une évolution spectaculaire sous Rituximab.

## Patient et observation

**Figure 1:**
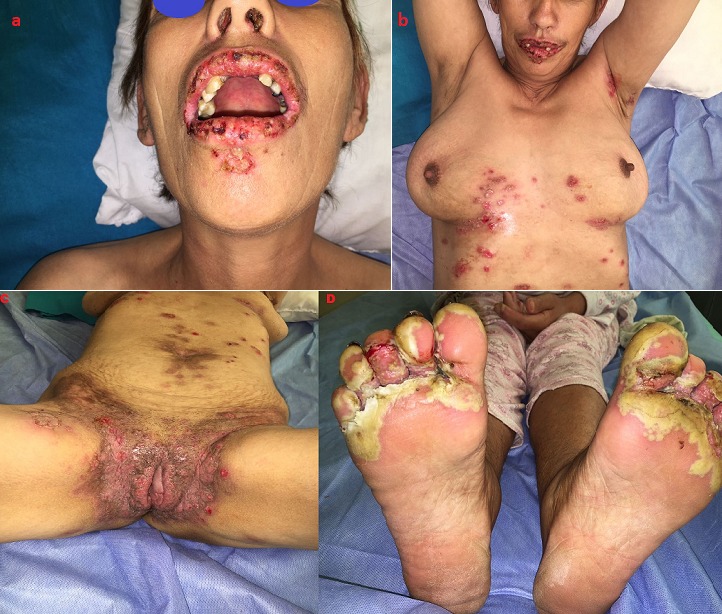
poussée du pemphigus végétant sous thérapies conventionnelles

**Figure 2:**
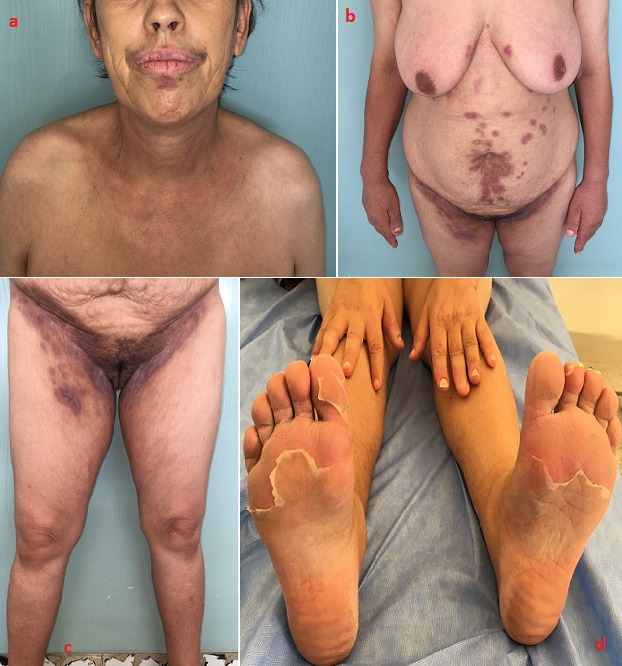
résolution complète des lésions après un mois de Rituximab

Il s'agit d'une patiente âgée de 42 ans, sans antécédents pathologiques notables. Elle avait consulté dans notre formation pour prise en charge de lésions végétantes siégeant au niveau des grands plis ainsi qu'au niveau des mains et des pieds évoluant depuis 2 ans, avec aggravation depuis 6 mois. L'examen dermatologique avait objectivé la présence de multiples placards végétants prédominant au niveau des plis axillaires, des plis inguinaux débordant sur le périnée, la région anale et l'ombilic. Ces placards étaient bien limités, à bord irréguliers avec des lésions similaires au niveau palmo plantaire, ainsi qu'une chéilite érosive et des érosions endobuccale. On avait réalisé une biopsie cutanée avec immunofluorescence directe qui avait confirmé le diagnostic du pemphigus végétant. La patiente avait été mise sous corticothérapie orale à la dose de 2mg/kg/jr et Azathioprine à la dose de 2mg/kg/jr avec légère amélioration durant le 1er mois, puis récidive Figure 1. En raison de l'absence de réponse thérapeutique avec des moyens agressifs, ainsi que la nécessité d'une thérapie plus efficace pour notre patiente, nous avons suggéré un essai avec Rituximab (RTX) basé sur des cas favorables rapportés dans la littérature. Le schéma posologique était de 375 mg/m^2^ en perfusions hebdomadaires pendant un mois. L'évolution a été marquée par la régression des plaques hypertrophiques en environ 1 mois, ne laissant qu'un léger érythème et une pigmentation post-inflammatoire résiduelle Figure 2. Le recul sans récidive est de 1 an.

## Discussion

Le pemphigus végétant est une variété rare de pemphigus, qui ne représente que 2% de ce trouble [1]. Son diagnostic est confirmé par l'examen histologique, qui montre une hyperplasie épidermique, une acantholyse épidermique profonde et une pustulose épidermique à polynucléaires neutrophiles et éosinophiles. L'immunofluorescence directe objective des dépôts d'IgG et de C3 en « mailles de filet » au niveau de l'épiderme. La physiopathologie du pemphigus végétant est mal connue [2]. Les auto-anticorps IgG circulants reconnaissent, comme dans le pemphigus vulgaire, la desmogléine 3. D'autres cibles antigéniques sont possibles, comme la desmogléine 1 et la périplakine. Mais d'autres facteurs, tels que des cytokines, joueraient un rôle dans le développement de la prolifération épidermique et le chimiotactisme des polynucléaires. En ce qui concerne le traitement, les corticostéroïdes topiques et/ou oraux sont recommandés comme traitement de première intention dans les documents de consensus actuels et les recommandations thérapeutiques, en particulier dans le pemphigus vulgaire, qui peuvent être étendus au sous-groupe pemphigus végétant [3-5]. Il est largement admis que leur utilisation a significativement réduit la morbidité et la mortalité chez ces patients. Néanmoins, les effets secondaires et les complications associés à l'utilisation de stéroïdes ont conduit à l'utilisation d'agents immunosuppresseurs tels que l'Azathioprine, la Cyclosporine, le Méthotrexate, le Cyclophosphamide et le Mycophénolate mofétil. L'utilisation d'immunoglobulines intraveineuses dans des cas réfractaires, et d'anti CD 20 notamment le RTX [6, 7], a entraîné une révolution complète dans la prise en charge de ce trouble. Malgré cela, le schéma posologique de RTX reste controversé. Ainsi, certains cliniciens soutiennent le régime lymphome (375 mg/m^2^ par semaine pendant quatre semaines), tandis que d'autres préfèrent celui utilisé dans l'arthrite rhumatoïde (1 g en deux séances à 15 jours d'intervalle), ou un système mixte d'induction. Le régime lymphome a été préconisé pour notre cas du même avis que Leventhal et Sanchez [8], avec une évolution spectaculaire et des effets secondaires moindres.

## Conclusion

La forme végétante du pemphigus est une forme exceptionnelle altérant la qualité de vie personnelle et professionnelle des patients. Heureusement maitrisée grâce à l'utilisation de la corticothérapie et certains immunosuppresseurs voir actuellement la révolution Rituximab. Néanmoins, a une époque où la pharmacoéconomie est au premier plan, il est essentiel d'obtenir les traitements les plus efficaces au prix le plus bas possible surtout dans notre contexte, et toujours avec le profil de risque le plus avantageux.

## Conflits d’intérêts

Les auteurs ne déclarent aucun conflit d'intérêts.
